# Molecular Characterization Analysis of Prevalent Infectious Bronchitis Virus and Pathogenicity Assessment of Recombination Strain in China

**DOI:** 10.3389/fvets.2022.842179

**Published:** 2022-07-22

**Authors:** Zhiqiang Wu, Huanxin Fang, Zhouyi Xu, Jiamin Lian, Zi Xie, Zhanxin Wang, Jianpin Qin, Benli Huang, Keyu Feng, Xinheng Zhang, Wencheng Lin, Hongxin Li, Weiguo Chen, Qingmei Xie

**Affiliations:** ^1^Heyuan Branch, Guangdong Provincial Laboratory of Lingnan Modern Agricultural Science and Technology, Guangdong Provincial Key Lab of Agro-Animal Genomics and Molecular Breeding, Key Laboratory of Chicken Genetics, Breeding and Reproduction, Ministry of Agriculture, College of Animal Science, South China Agricultural University, Guangzhou, China; ^2^Wen's Group Academy, Wen's Foodstuffs Group Co., Ltd., Xinxing, China; ^3^Guangdong Engineering Research Center for Vector Vaccine of Animal Virus, Guangzhou, China; ^4^South China Collaborative Innovation Center for Poultry Disease Control and Product Safety, Guangzhou, China; ^5^Key Laboratory of Animal Health Aquaculture and Environmental Control, College of Animal Science, South China Agricultural University, Guangzhou, China

**Keywords:** infectious bronchitis virus, *S1* gene, recombination, mutation, pathogenicity

## Abstract

Avian coronavirus infectious bronchitis virus (IBV) is a respiratory pathogen of chickens, resulting in severe economic losses in the poultry industry. This study aimed to monitor and isolate the molecular identity of IBV in broiler flocks with respiratory symptoms in eight provinces of China. In total, 910 samples (oropharyngeal and cloacal mixed swabs) from broiler flocks showed IBV positive rates of 17.6% (160/910) using PCR assay. Phylogenetic analysis of the complete *S1* genes of 160 IBV isolates was performed and revealed that QX-type (GI-19), TW-type (GI-7), 4/91-type (GI-13), HN08-type (GI-22),TC07-2-type (GVI-1), and LDT3-type (GI-28) exhibited IBV positive rates of 58.15, 25, 8.12, 1.86, 5.62, and 1.25%. In addition, recombination analyses revealed that the four newly IBV isolates presented different recombination patterns. The CK/CH/JS/YC10-3 isolate likely originated from recombination events between strain YX10 (QX-type) and strain TW2575-98 (TW-type), the pathogenicity of which was assessed, comparing it with strain GZ14 (TW-type) and strain CK/CH/GD/JR07-7 (QX-type). The complete *S1* gene data from these isolates indicate that IBV has consistently evolved through genetic recombination or mutation, more likely changing the viral pathogenicity and leading to larger outbreaks in chick populations, in China.

## Introduction

Infectious bronchitis (IB) is a highly contagious and widespread viral disease of economic significance, affecting severely the global poultry industry (1). Infectious bronchitis virus (IBV), a member of the γ*-Coronavirus* genus in the *Coronaviridae* family, is one of the etiologic agents of upper respiratory disease in chickens of all ages and varieties, as well as it causes urogenital illness, which is characterized by gout or nephritis (2, 3). IBV is prone to genetic evolution through the gradual accumulation of mutations and drastic recombination events. Currently, lots of new IBV variants have been continuously emerging, while vaccines have been created and inoculated worldwide. In China, based on *S1* gene phylogenetic analysis, IBV is mainly divided into sever genotypes, including QX-type (GI-19), TW-type (GI-7), 4/91-type (GI-13), Mass-type (GI-1), HN08-type (GI-22), TC07-2-type (GVI-1), and LDT3-type (GI-28) (4–6). QX-type strains, with changes in geographical distribution and tissue tropism (7), have been epidemic and predominant in poultry flocks since their initial emergence in 1996 (8, 9). Notably, TW-type and 4/91-type have been identified more frequently in recent years (10). The following are susceptible factors for the continuous emergence of new IBVs: (a) in the process of viral replication, its genomes are prone to insertion, deletion, and substitution; (b) natural recombination occurs randomly in various viral RNAs; (c) live attenuated or inactivated commercial IBV vaccines show little or no cross-protection against different genotype strains and even carry the risk of recombination between vaccine strains and wild type strains.

In this brief report, we analyzed the molecular characterization of 160 IBV isolates from eight provinces in China in 2020–2021. It provides detailed evidence of the severe prevalence of IBV in China, and they also provide a reminder that more scientific vaccine strategies are needed, including strain selection and cross-protection assessment to provide broad protection against different IBVs.

## Materials and Methods

### Ethics Statement

The use of animals in this study was approved by the South China Agricultural University Committee for Animal Experiments [approval ID: SYXK (Guangdong) 2019-0136]. All study procedures and animal care activities were conducted following the national and institutional guidelines for the care and use of laboratory animals.

### The Prevalence of IBV in Chick Flock

Between Sept 2020 and July 2021, several chick flocks, having developed upper respiratory symptoms, including coughing, wheezing, rales, and nasal discharge, were suspected of being infected with IBV. A total of 910 oropharyngeal and cloacal mixed swab samples were collected from the suspected chick flocks from eight provinces in mainland China (Fujian, Guangdong, Guangxi, Yunnan, Hunan, Hubei, Jiangsu, and Zhejiang). The swab specimens were stored in 500 μL of 1 mM phosphate-buffered saline (PBS) containing 200 U/mL of penicillin and 200 μg/mL of streptomycin, and vortexed for 20 min to prepare the virus solution for testing. The total RNA was extracted by using the RaPure Viral RNA/DNA Kit (Magen, Guangdong, China). The viral RNA was measured by quantitative reverse transcription PCR (qRT-PCR) according to the following protocol (10) using the HiScript® II One Step qRT-PCR Probe Kit (Vazyme, Nanjing, China), with the primer pairs designed to target the gene RNA nucleotide sequence (Accession: AF093793.1). The reaction conditions were as follows: 50°C for 5 min, 95°C for 30 s, and 45 cycles of 95°C for 5 s and 60°C for 34 s on the Applied Biosystems™ 7500 (Thermofisher, Beijing, China). The positive judgment value for this assay is between 15 and 35 cycle thresholds (**CT**) per 2 μL RNA template. The sequences of primers and probes used were as follows: IBV-F (5′-CCTCTAAGGGCTTTTGAG-3′), IBV-R (5′-GTCACTGTCTATTGTATGTC-3′), and IBV-P (5′-FAMCACCACCAGAACCTGTCACCTC BHQ1-3′).

### IBV Isolation and *S1* Gene Sequencing

Special pathogen free (SPF) embryonated eggs were purchased from Guangdong DHN Poultry and Egg Products Co. Ltd. (Yunfu, Guangdong, China). After the positive swab samples were vortexed shortly and centrifuged at 750 × *g* for 5 min at 4°C, the supernatants (0.2 mL) were collected and serially diluted 10-fold from 10^−1^ to 10^−3^ in PBS, which inoculate into 9-day-old SPF chicken embryonated eggs respectively with 0.2 mL by the allantoic cavity route. Infectious allantoic fluid was harvested from the embryos at 2–3 days post-inoculation and the viral RNA was determined as previously described. Next, specific primer pairs targeting the complete IBV *S1* gene were designed for reverse transcription polymerase chain reaction (**RT-PCR**). The thermal cycling (Bio-Rad, UK) parameters were as follows: 94°C for 1 min, and 30 cycles of 94°C for 30 s, 60°C for 30 s, and 72°C for 1.5 min. The complete *S1* gene, ~1,640 bp, was amplified using the PrimeScript™ RT-PCR Kit (Takara, Japan), and its products were separated on agarose gel and purified using a QIAquick Gel Extraction Kit (Qiagen, USA). The purified PCR products were cloned into a pMD™19-T vector (Takara, Japan) and then sequenced by BGI Genomics Co., Ltd. The sequences of the primers used were as follows: S1-F (5′-AACTGAACAAAAGACCGACT-3′), S1-R (5′-CAAAACCTGCCATAACTAACA-3′) (11).

### Complete *S1* Gene Phylogenetic and Recombination Analysis

The sequencing was performed using DNAStar Lasergene software (DNAStar, Madison, WI, USA). Additional publicly available complete *S1* gene sequences, obtained from GenBank, were aligned by the MEGA (version 7.0) ClustalW algorithm and the Test Neighbor-Joining Tree for designing the IBV *S1* phylogenetic tree. A total of 1,000 bootstraps were calculated. Finally, the tree was visualized using Interactive Tree Of Life (iTOL) web-based tool (http://itol.embl.de/). All of the reference complete IBV *S1* sequences representing all currently recognized types and subtypes used in the present study are listed in the Supplementary Material. Furthermore, the potential recombination events assessed by the Recombination Detection Program 4.0 (RDP 4.0, version 4.96) with strong *P*-values (<10–20), were further investigated by similarity plot and bootscan analyses implemented in Simplot 3.5.1.

### IBV Isolates Pathogenicity Test

A total of 30 ten-day-old SPF chickens were purchased from Guangdong DHN Poultry and Egg Products Co. Ltd. (Yunfu, Guangdong, China) and housed in the isolators and randomly divided into four groups. The GZ14 strain (TW-type) and strain CK/CH/GD/JR07-7 (QX-type) was isolated and saved in the laboratory. Subsequently, we inoculated the chicks intranasally with 10^6^ EID_50_ per 0.2 mL of GZ14 strains, CK/CH/GD/JR07-7 strains and CK/CH/JS/YC10-3 isolates respectively and used the same volume of sterile allantoic fluid for the control group. Postmortem was carried out 7 dpi, and the kidney and air sac lesions of the chickens were scored as previously described (12–14) (Tables 1, 2).

**Table 1:** Scoring of kidney lesions in chickens.

**Assigned score**	**Kidney lesions**
0	Normal, no lesions
1	Swollen and pale
2	Swollen with visible urates
3	Large swelling, pale with tubules, and ureters distended with urates
4	Nephritis

**Table 2:** Scoring of air sac lesions in chickens.

**Assigned score**	**Air sac lesions**
0	Normal, clean, thin, and transparent
1	Slightly thickened and slightly turbid, or individual local white exudate
2	Grayish white exudate in several areas of the air sac, moderate sac thickening
3	Majority of the air sacs fully covered with yellow white caseous exudate and obvious air sac thickening
4	Serious air sac lesions with thick white exudate on thoracic cavity and abdominal cavity

### Statistical Analysis

The gross lesion scores of kidney and air arc were analyzed using the one-way ANOVA followed by Tukey's multiple comparison tests. A *p*-value of <0.05 was considered a statistically significant difference (^*^), *p* < 0.01 was considered a moderately significant difference (^**^), and *p* < 0.001 was considered a highly significant difference (^***^).

## Results

### The Prevalence of IBV

**Figure 1:**
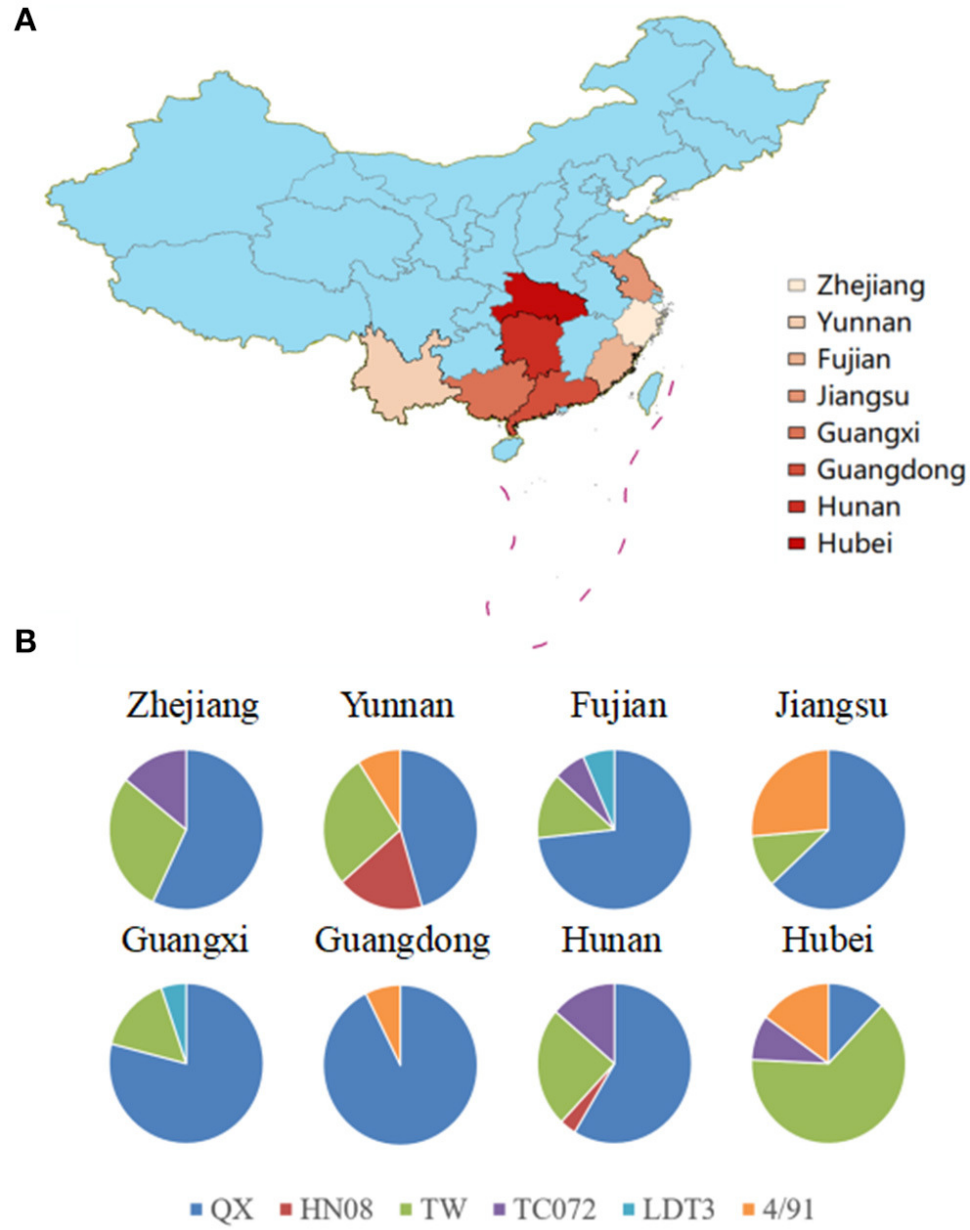
**(A)** Map of China with IBV strain prevalence in eight provinces in China where samples were collected. The map was created using ArcGis and the darker the color, the higher the level of positive rates of IBV specimens. **(B)** Different IBV strain prevalence rates in eight provinces. The graphs show the distribution of positive specimens for genotypes of IBV (QX in blue, HN08 in red, TW in green, TC072 in purple, LDT3 in purple, and 4/91 in orange).

Previous investigations have suggested that IBV will induce respiratory system and kidney disease in chickens (15, 16). In the present study, we collected swabs from chick flocks with suspected respiratory virus infections from eight provinces and analyzed them using RT-qPCR. In the examined flocks showing respiratory symptoms, 17.6% (160 out of 910) were infected with IBV. The most abundant strains were detected in the province of Hebei (Figure 1A). A total of 160 IBV strains were isolated and comparative analysis of the *S1* gene revealed that QX-type, TW-type, 4/91-type, HN08-type, TC07-2-type, and LDT3-type exhibited IBV positive rates of 58.15, 25, 8.12, 1.86, 5.62, and 1.25% (Table 3), respectively. Among them, the QX-type was isolated in 73.3% of chicks infected in Fujian, 78.9% in Guangxi, 92.5% in Guangdong, 50% in Yunnan, 58.6% in Hunan, 63.2% in Jiangsu, and 57.1% in Zhejiang. In addition, TW-type threatened 63.6% of infected chicks in the province of Hubei, and Mass-type was not discovered (Figure 1B).

**Table 3:** The distribution of 160 IBV isolates.

	**Fujian**	**Guangxi**	**Guangdong**	**Yunnan**	**Hunan**	**Hubei**	**Jiangsu**	**Zhejiang**
QX-type	11	15	25	5	17	4	12	4
HN08-type				2	1			
TW-type	2	3		3	7	21	2	2
TC072-type	1				4	3		1
LDT3-type	1	1						
4/91-type			2	1		5		5
Total	15	19	27	11	29	33	14	12

### The Genetic Evolution and Recombination of IBV

**Figure 2:**
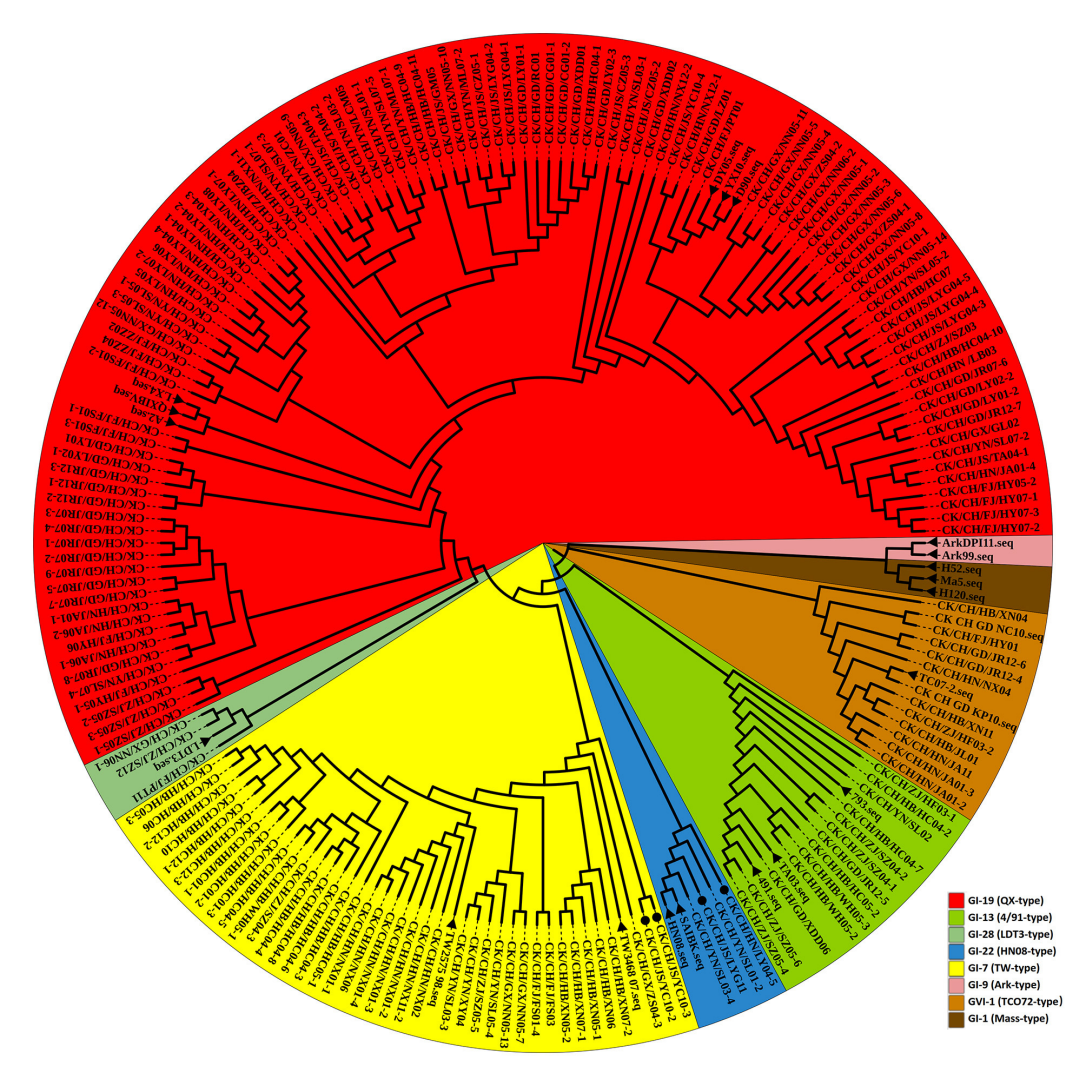
Phylogenetic analysis of the IBV *S1* gene. Circle tree: IBV isolates from Sept 2020 to July 2021 in China can be divided into six clusters. The reference strains are marked by a black solid triangle (▴). Newly recombinant strains are indicated with black solid circles (•). Other strains without a sign were excluded from this study.

The nucleotide sequence of the complete *S1* gene of isolates was aligned to relevant sequences that represent different IBV genotypes available in GenBank using MEGA 7.0. According to neighbor-joining tree phylogenetic analysis (Figure 2).

**Figure 3:**
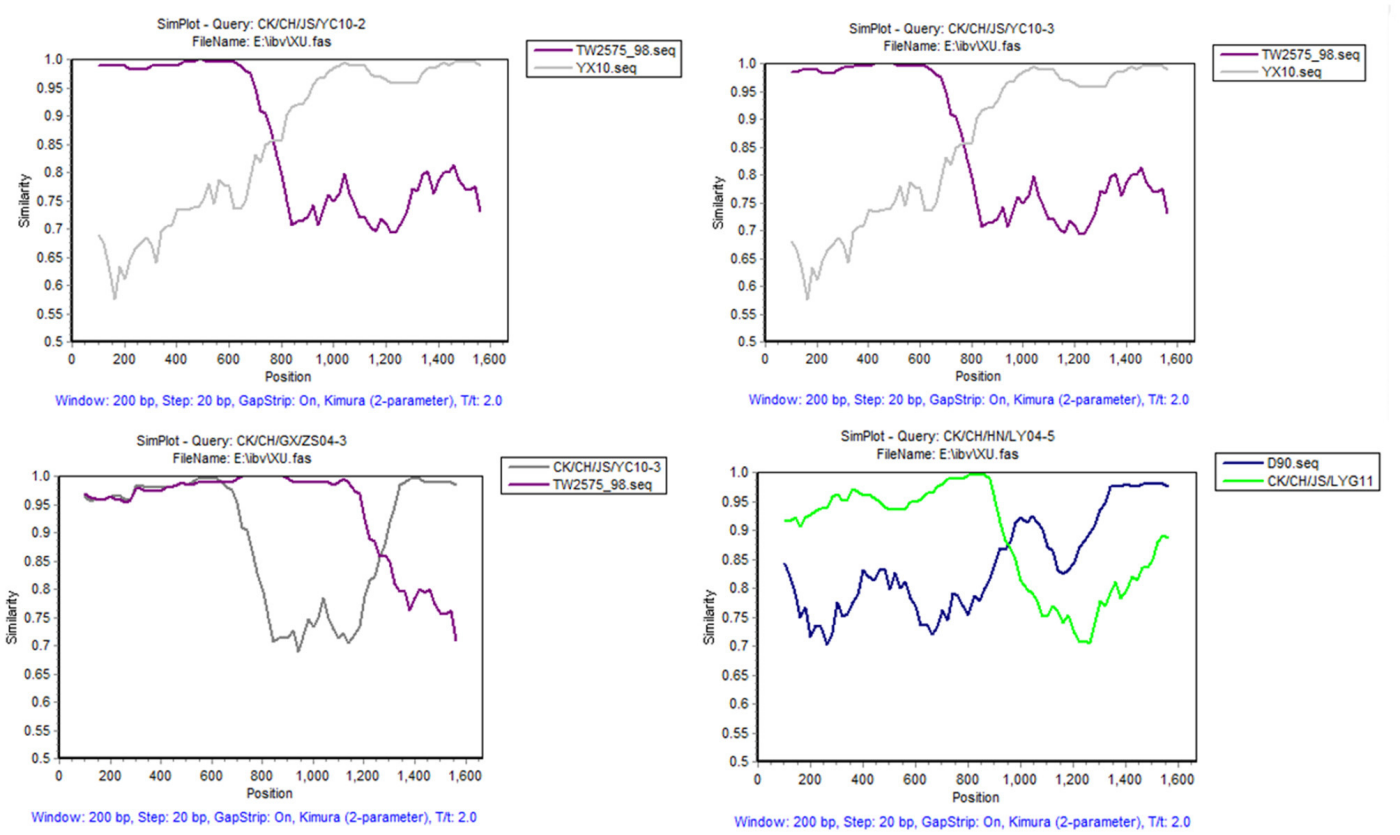
SimPlot analysis of recombination events of strains CK/CH/JS/YC10-2, CK/CH/JS/YC10-3, CK/CH/GX/ZS04-3, and CK/CH/HN/LY04-5. Strain CK/CH/JS/YC10-2 appears to be recombinant with strain YX10 as the major parent and TW2575-98 as the minor parent. Strain CK/CH/JS/YC10-3 appears to be recombinant with strain YX10 as the major parent and TW2575-98 as the minor parent. Strain CK/CH/GX/ZS04-3 appears to be recombinant with strain TW2575-98 as the major parent and CK/CH/JS/YC10-3 as the minor parent. Strain CK/CH/HN/LY04-5 appears to be recombinant with strain D90 as the major parent and CK/CH/JS/LYG11 as the minor parent.

To reveal possible recombination events, the complete *S1* genes of 20 reference strains were used as putative parent strains and recombination detection was conducted with RDP 4.0. Using at least five detection methods, the results suggest that four isolates' *S1* genes are possibly recombinant strains, with *p*-values of ≤ 1 × 10^−6^ (Table 4). As shown in Figure 3, strain CK/CH/JS/YC10-2 appears to be recombinant with strain YX10 as the major parent and TW2575-98 as the minor parent, with recombination breakpoints mapping to positions 747 (beginning breakpoint) and 1673 (ending breakpoint). Strain CK/CH/JS/YC10-3 appears to be recombinant with strain YX10 as the major parent and TW2575-98 as the minor parent, with recombination breakpoints mapping to positions 757 (beginning breakpoint) and 1674 (ending breakpoint). Strain CK/CH/GX/ZS04-3 appears to be recombinant with strain TW2575-98 as the major parent and CK/CH/JS/YC10-3 as the minor parent, with recombination breakpoints mapping to positions 1252 (beginning breakpoint) and 1672 (ending breakpoint). Strain CK/CH/HN/LY04-5 appears to be recombinant with strain D90 as the major parent and CK/CH/JS/LYG11 as the minor parent, with recombination breakpoints mapping to positions 88 (beginning breakpoint) and 948 (ending breakpoint).

**Table 4:** Information on recombination events.

**Breakpoint position**			**Major parent** ^**a**^			**Minor parent** ^**b**^			**Detection methods**							
**Strain**	**Begin**	**End**	**Strain**	**Genotype**	**Similarity**	**Strain**	**Genotype**	**Similarity**	*P*-value^*^	**R**	**G**	**B**	**M**	**C**	**S**	**T**
CK/CH/JS/YC10-2	747	1,673	YX10	QX-type	97.0%	TW2575-98	TW-type	99.0%	1.68 × 10^−33^	+	+	+	+	+	+	+
CK/CH/JS/YC10-3	757	1,674	YX10	QX-type	97.1%	TW2575-98	TW-type	98.9%	1.68 × 10^−33^	+	+	+	+	+	+	+
CK/CH/GX/ZS04-3	1,252	1,672	TW2575-98	TW-type	99.2%	CK/CH/JS/YC10-3	Unknown	98.6%	9.60 × 10^−21^	+	+	+	+	+	+	+
CK/CH/HN/LY04-5	88	948	D90	QX-type	94.2%	CK/CH/JS/LYG11	Unknown	96.2%	4.86 × 10^−14^	+	+	+	+	-	+	+

*Major parent^a^ = Sequence closely related to the transferred fragment in the recombinant*.

*Minor parent^b^ = Sequence most closely related to the sequence surrounding the transferred fragment*.

* *P-value of RDP method*.

*R, RDP; G, GENECONV; B, Bootscan; M, Maxchi; C, Chimera; S, SiScan; T, Tree Topo*.

### IBV Isolate Pathogenicity Test

Based on the viral recombination results, the CK/CH/JS/YC10-3 isolate, which was formed by the major parent strain YX10 (QX-type) and the minor parent strain TW2575-98 (TW-type), was used for the exposure tests.

**Figure 4:**
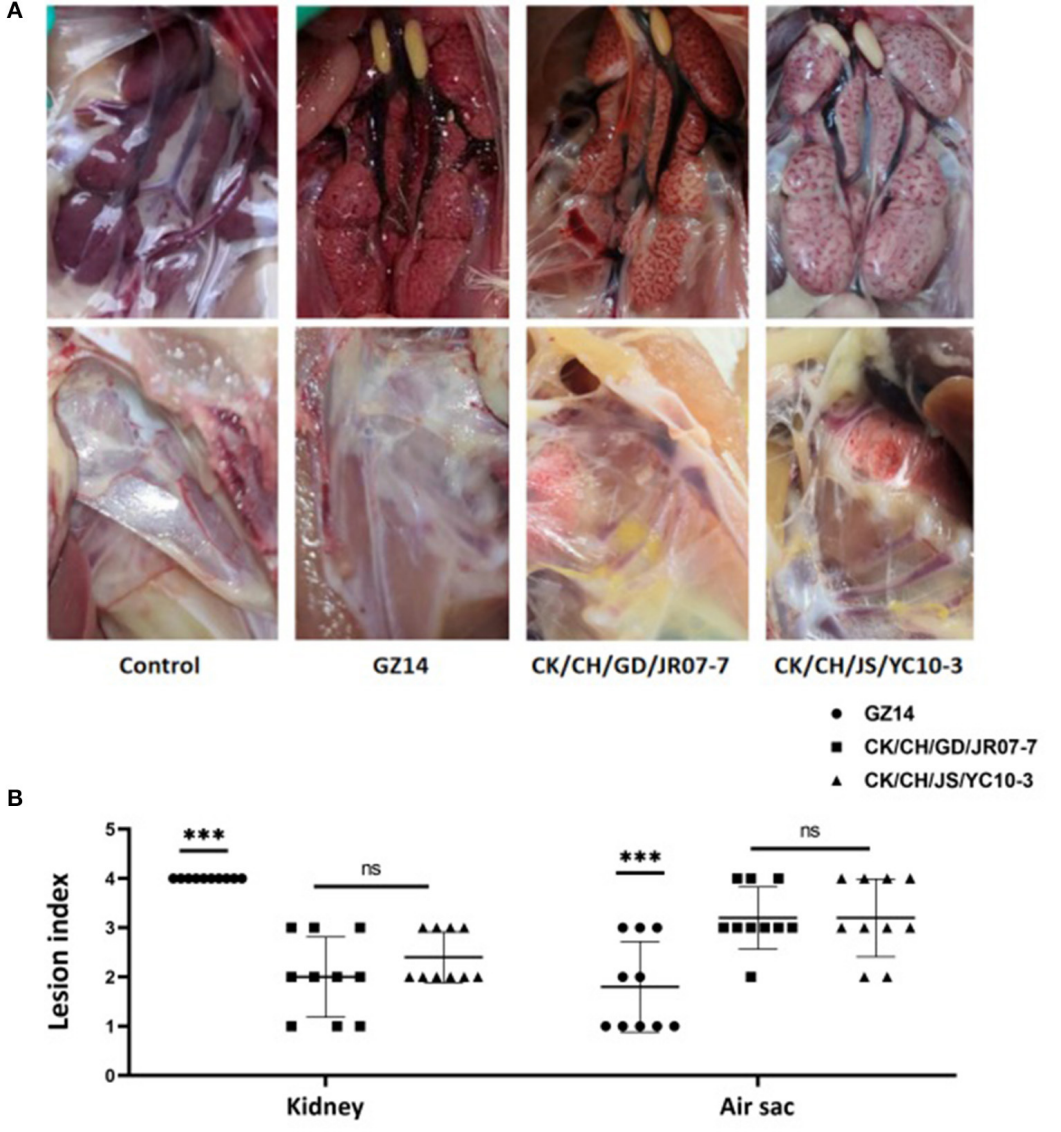
**(A)** Gross lesions from experimentally exposed chicks. Control group: No gross lesions in the kidneys and air sacs are clean, thin and transparent; GZ14: Nephritis, air sac turbidness; CK/CH/JS/YC10-3: Swollen kidney with urate, yellow white caseous exudate in air sacs. **(B)** Gross lesion scores of kidney and air sac by exposing with strain GZ14, CK/CH/GC/JR07-7, and CK/CH/JS/YC10-3. Each lesion's score independently reflects each chick's gross lesions. Black solid circles indicate the GZ14 group, black solid circles the CK/CH/GC/JR07-7 group, and black solid triangle indicate the CK/CH/JS/YC10-3 group. Using one-way ANOVA followed by Tukey's multiple comparison test, strain GZ14 and CK/CH/JS/YC10-3 exhibited a highly significant difference (***) in kidneys and air sacs with *p* < 0.001. Strain CK/CH/GC/JR07-7 and CK/CH/JS/YC10-3 exhibited non-significant (ns) with *p* > 0.05.

Postmortem, all of the birds exposed to strain GZ14 showed obvious lesions in the kidneys, in which they were mainly preformed nephritis, as well as different degrees of air sac turbidness. In contrast, the birds with strain CK/CH/GD/JR07-7 or the CK/CH/JS/YC10-3 isolate generally showed swollen kidneys and obviously thickened air sacs covered with yellow white caseous exudate. No clinical symptoms were observed in the chickens of the control group (Figure 4A). In addition, gross lesions in the air sacs and kidneys were scored and recorded (Figure 4B).

## Discussion

Attention has been paid to poultry respiratory system diseases worldwide in recent years. High morbidity rate and reduced egg and meat production are caused by respiratory tract infections, which are of paramount importance in the poultry industry. The potential pathogens induce high mortality rates, growth retardation, and high cost of medications, all of which decrease net income (17, 18). The etiology of respiratory infections is an ongoing exploration, with the majority of the studies have focused on chronic infections and various pathogen co-infections, including infectious bronchitis virus (IBV), Newcastle disease virus (NDV), low pathogenic avian influenza virus (LPAIV) H9N2, *Escherichia coli, Staphylococcus aureus, Mycoplasma gallisepticum* (MG), and *Mycoplasma synoviae* (MS); these pathogens can cause respiratory diseases independently, or together (19).

In this research, we focused on the impact of IBV infection in chickens in China. Throughout eight provinces, the situation of IBV prevalence is still serious, even though the chickens have been immunized with commercial live attenuated or inactivated IBV vaccine. IBV strains were successfully detected and isolated from Oropharyngeal and cloacal swabs of chick flocks with target symptoms. Based on phylogenetic analysis, the current prevalent strains can be distributed into six clusters, revealing a high genetic variety and potential pathogenicity complexity among IBV strains emerging in China. Specifically, QX-type strains have played a predominant role and TW-type strains are increasingly emerging. In addition, recombination strains were also identified. Furthermore, pathogenic assessment of selected strains was also performed by challenging 10-day-old SPF chickens. Our research result revealed that recombinant CK/CH/JS/YC10-3 isolate, formed by a major parent QX-type strain and a minor parent TW-type strain, presented lesion of air sacs covered with yellow white caseous exudate and obvious air sac thickening which similar to CK/CH/GC/JR07-7 strain (QX-type). However, GZ14 strain (TW-type) showed its damage mainly with acute nephritis.

IBV, a single molecule RNA virus, can mutate frequently for complex mechanisms, leading to extensive antigenic variation (20). Recombination events are the important factors of generating new strains, and sometimes, random mutations also involve this procedure (21). The genetic alterations found in the *S1* gene will urge the corresponding S1 protein to mutate in IBV variants (22). Eventually, it might contribute to differences in viral replication, pathogenicity, antigenicity, immunogenicity, and tissue tropism (23). In this study, analysis of the complete *S1* gene of these isolates definitively proved that IBV is continuously undergoing these processes, and simultaneity its biological characteristics has changed probably. In productive activities, chick flocks in China were received already the IBV H120, 4/91, or QX vaccine in 1-day-old. However, it also happened high positive rate of wild type virus infection. The caveat is that the recombination strain hardly shows weaker virulence than the classical strain, which is a significant challenge for the effectiveness of the vaccine. It is difficult for the commercial vaccine to make a difference in cross-protection against various IBVs.

It is particularly notable here that controlling IB in China remains a challenging task due to the co-circulation of numerous IBV genotypes and the increasing isolation of novel strains (24). Therefore, it is necessary to continuously monitor the different genotypes or lineages of IBV and make effort to decrease IBVs co-circulation so that the risks of IBV vaccination failure can be avoided and new effective vaccines can be developed. Nowadays, multiple pathogen co-infections causing respiratory diseases are common in production. Even though we did not detect whether all of the specimens carry other pathogens in this study, it is worth considering that LPAIV or MG possibly plays a role. Therefore, the synergy between IBV to other respiratory etiologic agents should be explored as soon as possible, as it is of considerable significance for prevention. This present pathogenic testing of strain GZ14, CK/CH/GC/JR07-7, and CK/CH/JS/YC10-3 laid the foundation for further research.

## Data Availability Statement

The original contributions presented in the study are included in the article/Supplementary Materials, further inquiries can be directed to the corresponding author.

## Ethics Statement

The animal study was reviewed and approved by South China Agricultural University.

## Author Contributions

Conceptualization, project administration, and funding acquisition: QX. Methodology: HF and ZWu. Software, writing—original draft preparation, and formal analysis: HF. Validation: ZWu, ZXu, and JL. Investigation: ZWu and ZXu. Resources: ZWu. Data curation: HF and ZXi. Writing—review and editing: ZWu and ZWa. Visualization: XZ. All authors have read and agreed to the published version of the manuscript.

## Conflict of Interest

ZWu, HF, Zxu, ZWa, and JQ were employed by Wen's Group Academy, Wen's Foodstuffs Group Co., Ltd. The remaining authors declare that the research was conducted in the absence of any commercial or financial relationships that could be construed as a potential conflict of interest.

## Publisher's Note

All claims expressed in this article are solely those of the authors and do not necessarily represent those of their affiliated organizations, or those of the publisher, the editors and the reviewers. Any product that may be evaluated in this article, or claim that may be made by its manufacturer, is not guaranteed or endorsed by the publisher.

## Abbreviations

IBV: infectious bronchitis virus
IB: infectious bronchitis
+ss: single-stranded and positive-sense
kb: kilobases
S: spike glycoprotein
E: envelope protein
M: membrane glycoprotein
N: nucleocapsid protein
PBS: phosphate-buffered saline
RT-qPCR: quantitative reverse transcription polymerase chain reaction
CT: cycle thresholds
SPF: special pathogen free
RT-PCR: reverse transcription polymerase chain reaction
RDP: the recombination detection program
MRs: mortality rates
NDV: newcastle disease virus
LPAIV: low pathogenic avian influenza virus
MG: mycoplasma gallisepticum
MS: mycoplasma synoviae.
