# Supplementary material for: Molecular Characterization Analysis of Prevalent Infectious Bronchitis Virus and Pathogenicity Assessment of Recombination Strain in China
Source: Front Vet Sci. 2022 Jul 22;9:842179. doi: 10.3389/fvets.2022.842179 (PMC9356287; doi:10.3389/fvets.2022.842179)
Supplement: Supplementary file 1 [file Data_Sheet_1.docx]

**Supplementary Table 1** IBV strains isolated from flocks in China.

| **Strain Name** | **Origin** | **genotype** | **lineage** | **Accession No.** |
| --- | --- | --- | --- | --- |
| CK/CH/FJ/FS01-2 | Fujian | QX-type | GI-19 | ON149091 |
| CK/CH/FJ/FS01-3 | Fujian | QX-type | GI-19 | ON149092 |
| CK/CH/FJ/FS01-4 | Fujian | TW-type | GI-7 | ON149093 |
| CK/CH/FJ/FS03 | Fujian | TW-type | GI-7 | ON149094 |
| CK/CH/FJ/HY01 | Fujian | TC072-type | GVI-1 | ON149095 |
| CK/CH/FJ/HY05-1 | Fujian | QX-type | GI-19 | ON149096 |
| CK/CH/FJ/HY05-2 | Fujian | QX-type | GI-19 | ON149097 |
| CK/CH/FJ/HY06 | Fujian | QX-type | GI-19 | ON149098 |
| CK/CH/FJ/HY07-1 | Fujian | QX-type | GI-19 | ON149099 |
| CK/CH/FJ/HY07-2 | Fujian | QX-type | GI-19 | ON149100 |
| CK/CH/FJ/HY07-3 | Fujian | QX-type | GI-19 | ON149101 |
| CK/CH/FJ/PT01 | Fujian | QX-type | GI-19 | ON149102 |
| CK/CH/FJ/PT11 | Fujian | LDT3-type | GI-28 | ON149103 |
| CK/CH/FJ/ZZ02 | Fujian | QX-type | GI-19 | ON149104 |
| CK/CH/FJ/ZZ04 | Fujian | QX-type | GI-19 | ON149105 |
| CK/CH/GD/CG01-1 | Guangdong | QX-type | GI-19 | ON149106 |
| CK/CH/GD/CG01-2 | Guangdong | QX-type | GI-19 | ON149107 |
| CK/CH/GD/JR07-1 | Guangdong | QX-type | GI-19 | ON149108 |
| CK/CH/GD/JR07-2 | Guangdong | QX-type | GI-19 | ON149109 |
| CK/CH/GD/JR07-3 | Guangdong | QX-type | GI-19 | ON149110 |
| CK/CH/GD/JR07-4 | Guangdong | QX-type | GI-19 | ON149111 |
| CK/CH/GD/JR07-5 | Guangdong | QX-type | GI-19 | ON149112 |
| CK/CH/GD/JR07-6 | Guangdong | QX-type | GI-19 | ON149113 |
| CK/CH/GD/JR07-7* | Guangdong | QX-type | GI-19 | ON149114 |
| CK/CH/GD/JR07-8 | Guangdong | QX-type | GI-19 | ON149115 |
| CK/CH/GD/JR07-9 | Guangdong | QX-type | GI-19 | ON149116 |
| CK/CH/GD/JR12-1 | Guangdong | QX-type | GI-19 | ON149117 |
| CK/CH/GD/JR12-2 | Guangdong | QX-type | GI-19 | ON149118 |
| CK/CH/GD/JR12-3 | Guangdong | QX-type | GI-19 | ON149119 |
| CK/CH/GD/JR12-5 | Guangdong | 4/91-type | GI-13 | ON149121 |
| CK/CH/GD/JR12-7 | Guangdong | QX-type | GI-19 | ON149123 |
| CK/CH/GD/LY01 | Guangdong | QX-type | GI-19 | ON149124 |
| CK/CH/GD/LY01-1 | Guangdong | QX-type | GI-19 | ON149125 |
| CK/CH/GD/LY01-2 | Guangdong | QX-type | GI-19 | ON149126 |
| CK/CH/GD/LY02-1 | Guangdong | QX-type | GI-19 | ON149127 |
| CK/CH/GD/LY02-2 | Guangdong | QX-type | GI-19 | ON149128 |
| CK/CH/GD/LY02-3 | Guangdong | QX-type | GI-19 | ON149129 |
| CK/CH/GD/LZ01 | Guangdong | QX-type | GI-19 | ON149130 |
| CK/CH/GD/RC01 | Guangdong | QX-type | GI-19 | ON149131 |
| CK/CH/GD/XDD01 | Guangdong | QX-type | GI-19 | ON149132 |
| CK/CH/GD/XDD02 | Guangdong | QX-type | GI-19 | ON149133 |
| CK/CH/GD/XDD06 | Guangdong | 4/91-type | GI-13 | ON149134 |
| CK/CH/GX/NN05-1 | Guangxi | QX-type | GI-19 | ON149136 |
| CK/CH/GX/NN05-10 | Guangxi | QX-type | GI-19 | ON149137 |
| CK/CH/GX/NN05-11 | Guangxi | QX-type | GI-19 | ON149138 |
| CK/CH/GX/NN05-12 | Guangxi | QX-type | GI-19 | ON149139 |
| CK/CH/GX/NN05-13 | Guangxi | TW-type | GI-7 | ON149140 |
| CK/CH/GX/NN05-14 | Guangxi | QX-type | GI-19 | ON149141 |
| CK/CH/GX/NN05-2 | Guangxi | QX-type | GI-19 | ON149142 |
| CK/CH/GX/NN05-3 | Guangxi | QX-type | GI-19 | ON149143 |
| CK/CH/GX/NN05-4 | Guangxi | QX-type | GI-19 | ON149144 |
| CK/CH/GX/NN05-5 | Guangxi | QX-type | GI-19 | ON149145 |
| CK/CH/GX/NN05-6 | Guangxi | QX-type | GI-19 | ON149146 |
| CK/CH/GX/NN05-7 | Guangxi | TW-type | GI-7 | ON149147 |
| CK/CH/GX/NN05-8 | Guangxi | QX-type | GI-19 | ON149148 |
| CK/CH/GX/NN05-9 | Guangxi | QX-type | GI-19 | ON149149 |
| CK/CH/GX/NN06-1 | Guangxi | LDT3-type | GI-28 | ON149150 |
| CK/CH/GX/NN06-2 | Guangxi | QX-type | GI-19 | ON149151 |
| CK/CH/GX/ZS04-1 | Guangxi | QX-type | GI-19 | ON149152 |
| CK/CH/GX/ZS04-2 | Guangxi | QX-type | GI-19 | ON149153 |
| CK/CH/GX/ZS04-3 | Guangxi | TW-type | GI-7 | ON149154 |
| CK/CH/HB/HC01-1 | Hubei | TW-type | GI-7 | ON149155 |
| CK/CH/HB/HC01-2 | Hubei | TW-type | GI-7 | ON149156 |
| CK/CH/HB/HC01-3 | Hubei | TW-type | GI-7 | ON149157 |
| CK/CH/HB/HC04-10 | Hubei | QX-type | GI-19 | ON149159 |
| CK/CH/HB/HC04-11 | Hubei | QX-type | GI-19 | ON149160 |
| CK/CH/HB/HC04-2 | Hubei | 4/91-type | GI-13 | ON149161 |
| CK/CH/HB/HC04-3 | Hubei | TW-type | GI-7 | ON149162 |
| CK/CH/HB/HC04-4 | Hubei | TW-type | GI-7 | ON149163 |
| CK/CH/HB/HC04-5 | Hubei | TW-type | GI-7 | ON149164 |
| CK/CH/HB/HC04-6 | Hubei | TW-type | GI-7 | ON149165 |
| CK/CH/HB/HC04-7 | Hubei | 4/91-type | GI-13 | ON149166 |
| CK/CH/HB/HC04-8 | Hubei | TW-type | GI-7 | ON149167 |
| CK/CH/HB/HC04-9 | Hubei | QX-type | GI-19 | ON149168 |
| CK/CH/HB/HC05-1 | Hubei | TW-type | GI-7 | ON149169 |
| CK/CH/HB/HC05-2 | Hubei | 4/91-type | GI-13 | ON149170 |
| CK/CH/HB/HC05-3 | Hubei | TW-type | GI-7 | ON149171 |
| CK/CH/HB/HC06 | Hubei | TW-type | GI-7 | ON149172 |
| CK/CH/HB/HC07 | Hubei | QX-type | GI-19 | ON149173 |
| CK/CH/HB/HC10 | Hubei | TW-type | GI-7 | ON149174 |
| CK/CH/HB/HC12-1 | Hubei | TW-type | GI-7 | ON149175 |
| CK/CH/HB/HC12-2 | Hubei | TW-type | GI-7 | ON149176 |
| CK/CH/HB/HC12-3 | Hubei | TW-type | GI-7 | ON149177 |
| CK/CH/HB/JL01 | Hubei | TC072-type | GVI-1 | ON149178 |
| CK/CH/HB/WH05-1 | Hubei | TW-type | GI-7 | ON149179 |
| CK/CH/HB/WH05-2 | Hubei | 4/91-type | GI-13 | ON149180 |
| CK/CH/HB/WH05-3 | Hubei | 4/91-type | GI-13 | ON149181 |
| CK/CH/HB/XN04 | Hubei | TC072-type | GVI-1 | ON149182 |
| CK/CH/HB/XN05-1 | Hubei | TW-type | GI-7 | ON149183 |
| CK/CH/HB/XN05-2 | Hubei | TW-type | GI-7 | ON149184 |
| CK/CH/HB/XN06 | Hubei | TW-type | GI-7 | ON149185 |
| CK/CH/HB/XN07-1 | Hubei | TW-type | GI-7 | ON149186 |
| CK/CH/HB/XN07-2 | Hubei | TW-type | GI-7 | ON149187 |
| CK/CH/HB/XN11 | Hubei | TC072-type | GVI-1 | ON149188 |
| CK/CH/HN/JA01-1 | Hunan | QX-type | GI-19 | ON149189 |
| CK/CH/HN/JA01-2 | Hunan | TC072-type | GVI-1 | ON149190 |
| CK/CH/HN/JA01-3 | Hunan | TC072-type | GVI-1 | ON149191 |
| CK/CH/HN/JA01-4 | Hunan | QX-type | GI-19 | ON149192 |
| CK/CH/HN/JA06-1 | Hunan | QX-type | GI-19 | ON149193 |
| CK/CH/HN/JA06-2 | Hunan | QX-type | GI-19 | ON149194 |
| CK/CH/HN/JA11 | Hunan | TC072-type | GVI-1 | ON149195 |
| CK/CH/HN/LY04-1 | Hunan | QX-type | GI-19 | ON149196 |
| CK/CH/HN/LY04-2 | Hunan | QX-type | GI-19 | ON149197 |
| CK/CH/HN/LY04-3 | Hunan | QX-type | GI-19 | ON149198 |
| CK/CH/HN/LY04-4 | Hunan | QX-type | GI-19 | ON149199 |
| CK/CH/HN/LY04-5 | Hunan | HN08-type | GI-22 | ON149200 |
| CK/CH/HN/LY05 | Hunan | QX-type | GI-19 | ON149201 |
| CK/CH/HN/LY06 | Hunan | QX-type | GI-19 | ON149202 |
| CK/CH/HN/LY07-1 | Hunan | QX-type | GI-19 | ON149203 |
| CK/CH/HN/LY07-2 | Hunan | QX-type | GI-19 | ON149204 |
| CK/CH/HN/LY08 | Hunan | QX-type | GI-19 | ON149205 |
| CK/CH/HN/NX01-1 | Hunan | TW-type | GI-7 | ON149206 |
| CK/CH/HN/NX01-2 | Hunan | TW-type | GI-7 | ON149207 |
| CK/CH/HN/NX01-3 | Hunan | TW-type | GI-7 | ON149208 |
| CK/CH/HN/NX01-4 | Hunan | TW-type | GI-7 | ON149209 |
| CK/CH/HN/NX02 | Hunan | TW-type | GI-7 | ON149210 |
| CK/CH/HN/NX04 | Hunan | TC072-type | GVI-1 | ON149211 |
| CK/CH/HN/NX06 | Hunan | TW-type | GI-7 | ON149212 |
| CK/CH/HN/NX11-1 | Hunan | QX-type | GI-19 | ON149213 |
| CK/CH/HN/NX11-2 | Hunan | TW-type | GI-7 | ON149214 |
| CK/CH/HN/NX12-1 | Hunan | QX-type | GI-19 | ON149215 |
| CK/CH/HN/NX12-2 | Hunan | QX-type | GI-19 | ON149216 |
| CK/CH/HN_/LB03 | Hunan | QX-type | GI-19 | ON149217 |
| CK/CH/JS/CZ05-2 | Jiangsu | QX-type | GI-19 | ON149218 |
| CK/CH/JS/CZ05-3 | Jiangsu | QX-type | GI-19 | ON149219 |
| CK/CH/JS/LYG04-1 | Jiangsu | QX-type | GI-19 | ON149221 |
| CK/CH/JS/LYG04-2 | Jiangsu | QX-type | GI-19 | ON149222 |
| CK/CH/JS/LYG04-3 | Jiangsu | QX-type | GI-19 | ON149223 |
| CK/CH/JS/LYG04-4 | Jiangsu | QX-type | GI-19 | ON149224 |
| CK/CH/JS/LYG04-5 | Jiangsu | QX-type | GI-19 | ON149225 |
| CK/CH/JS/TA04-1 | Jiangsu | QX-type | GI-19 | ON149226 |
| CK/CH/JS/TA04-2 | Jiangsu | QX-type | GI-19 | ON149227 |
| CK/CH/JS/TA04-3 | Jiangsu | QX-type | GI-19 | ON149228 |
| CK/CH/JS/YC10-1 | Jiangsu | QX-type | GI-19 | ON149229 |
| CK/CH/JS/YC10-2 | Jiangsu | TW-type | GI-7 | ON149230 |
| CK/CH/JS/YC10-3* | Jiangsu | TW-type | GI-7 | ON149231 |
| CK/CH/JS/YC10-4 | Jiangsu | QX-type | GI-19 | ON149232 |
| CK/CH/ZJ/HF03-1 | Zhejiang | 4/91-type | GI-13 | ON149254 |
| CK/CH/ZJ/SZ04-1 | Zhejiang | 4/91-type | GI-13 | ON149257 |
| CK/CH/ZJ/SZ04-2 | Zhejiang | 4/91-type | GI-13 | ON149258 |
| CK/CH/ZJ/SZ05-4 | Zhejiang | 4/91-type | GI-13 | ON149263 |
| CK/CH/ZJ/SZ05-6 | Zhejiang | 4/91-type | GI-13 | ON149265 |
| CK/CH/YN/SL01-2 | Yunan | HN08-type | GI-22 | ON149237 |
| CK/CH/YN/SL02 | Yunan | 4/91-type | GI-13 | ON149238 |
| CK/CH/YN/SL03-2 | Yunan | QX-type | GI-19 | ON149240 |
| CK/CH/YN/SL03-3 | Yunan | TW-type | GI-7 | ON149241 |
| CK/CH/YN/SL03-4 | Yunan | HN08-type | GI-22 | ON149242 |
| CK/CH/YN/SL05-4 | Yunan | TW-type | GI-7 | ON149246 |
| CK/CH/YN/SL07-2 | Yunan | QX-type | GI-19 | ON149247 |
| CK/CH/YN/SL07-3 | Yunan | QX-type | GI-19 | ON149248 |
| CK/CH/YN/SL07-4 | Yunan | QX-type | GI-19 | ON149249 |
| CK/CH/YN/SL07-5 | Yunan | QX-type | GI-19 | ON149250 |
| CK/CH/YN/XY04 | Yunan | TW-type | GI-7 | ON149251 |
| CK/CH/ZJ/BZ04 | Zhejiang | QX-type | GI-19 | ON149253 |
| CK/CH/ZJ/HF03-2 | Zhejiang | TC072-type | GVI-1 | ON149255 |
| CK/CH/ZJ/SZ04-3 | Zhejiang | TW-type | GI-7 | ON149259 |
| CK/CH/ZJ/SZ05-1 | Zhejiang | QX-type | GI-19 | ON149260 |
| CK/CH/ZJ/SZ05-2 | Zhejiang | QX-type | GI-19 | ON149261 |
| CK/CH/ZJ/SZ05-3 | Zhejiang | QX-type | GI-19 | ON149262 |
| CK/CH/ZJ/SZ05-5 | Zhejiang | TW-type | GI-7 | ON149264 |

* These strains were selected to conduct assessment of pathogenic test.**Supplementary Table 2** IBV reference strains used in this study.

| **Strain** | **Origin** | **Genotype** | **Lineage** | **Accession No.** |
| --- | --- | --- | --- | --- |
| YX10 | China | QX-type | GI-19 | [JX840411](https://www.ncbi.nlm.nih.gov/nuccore/JX840411) |
| D90 | China | QX-type | GI-19 | [MF508703](https://www.ncbi.nlm.nih.gov/nuccore/MF508703) |
| DY05 | China | QX-type | GI-19 | [GQ265928](https://www.ncbi.nlm.nih.gov/nuccore/GQ265928) |
| A2 | China | QX-type | GI-19 | [AY043312](https://www.ncbi.nlm.nih.gov/nuccore/AY043312) |
| LX4 | China | QX-type | GI-19 | [AY338732](https://www.ncbi.nlm.nih.gov/nuccore/AY338732) |
| QXIBV | China | QX-type | GI-19 | [AF193423](https://www.ncbi.nlm.nih.gov/nuccore/AF193423) |
| 4/91 | UK | 4/91-type | GI-13 | [AF093794](https://www.ncbi.nlm.nih.gov/nuccore/AF093794) |
| TA03 | China | 4/91-type | GI-13 | [AY837465](https://www.ncbi.nlm.nih.gov/nuccore/AY837465) |
| 7/93 | UK | 4/91-type | GI-13 | [Z83979](https://www.ncbi.nlm.nih.gov/nuccore/Z83979) |
| HN08 | China | HN08-type | GI-22 | [GQ265940](https://www.ncbi.nlm.nih.gov/nuccore/GQ265940) |
| SAIBK | China | HN08-type | GI-22 | [DQ288927](https://www.ncbi.nlm.nih.gov/nuccore/DQ288927) |
| ArkDPI | USA | Ark-type | GI-9 | [EU418976](https://www.ncbi.nlm.nih.gov/nuccore/EU418976) |
| Ark99 | USA | Ark-type | GI-9 | [M99482](https://www.ncbi.nlm.nih.gov/nuccore/M99482) |
| H52 | Netherlands | Mass-type | GI-1 | [AF352315](https://www.ncbi.nlm.nih.gov/nuccore/AF352315) |
| H120 | Netherlands | Mass-type | GI-1 | [EU822341](https://www.ncbi.nlm.nih.gov/nuccore/EU822341) |
| Ma5 | USA | Mass-type | GI-1 | [AY561713](https://www.ncbi.nlm.nih.gov/nuccore/AY561713) |
| TW3468/07 | China | TW-type | GI-7 | [EU822336](https://www.ncbi.nlm.nih.gov/nuccore/EU822336) |
| TW2575/98 | China | TW-type | GI-7 | [DQ646405](https://www.ncbi.nlm.nih.gov/nuccore/DQ646405) |
| TC07-2 | China | TC07-2-type | GVI-1 | [GQ265948](https://www.ncbi.nlm.nih.gov/nuccore/GQ265948) |
| LDT3 | China | LDT3-type | GI-28 | [KR608272](https://www.ncbi.nlm.nih.gov/nuccore/KR608272) |
